# The other organs

**DOI:** 10.4102/ajod.v3i1.124

**Published:** 2014-04-23

**Authors:** Karen Lazar

**Affiliations:** 1 Division of Languages, Literacies and Literatures, School of Education, University of the Witwatersrand, South Africa

## Crossroad

I’m the right hemisphere of your brain
I operate across the middle
I issue commands to your left arm and it waves at me
I call to your left knee and it flexes up a step.

What mischief is this, what paradox of symmetry?

My neighbour the left hemisphere is a stern chap
Good with numbers and logic, organised.
I’m the one holding the paint brush and the flute
I splash colour and I warble.

Sometimes we squabble, or quibble over turf.
If he stores your first language, can I store your second?

If the dam bursts
If I drown in blood or gasp for oxygen when a clot blocks your highway,
your waving arm and flexing knee surrender to weakness, or worse.

I try, I really try, to bring what’s left of me to take over from what’s gone.
Resume my creating, my issuing of commands.
But sometimes my sullen tissue wins out
And, straining, I send you signals.
They lie like post unread.
I rail in frustration, hurl myself against the skull

When he over the fence, my neighbour, gets clotted or washed into failure
You grow mute, of limb and tongue.
He twists and tries to scream at me: DO SOMETHING
I try to help with his work but often I am blocked by the divide.

We watch at the cross-roads, thwarted traffic officers,
Side by side, but sequestrated.

## Lung

I’ve never understood
Why my neighbour your left arm doesn’t stir
But here I am peacefully inflating and deflating
In, out, in, out.
The work of decades but I never grow bored
Does a tide at sea grow apathetic?
Your bossy heart booms at me, also working overtime
But working nonetheless.
Yet limp is the arm, and useless the foot’s limp
stricken into stillness.
My willing bronchi, tiny trees or kelp
Wave at you in a soft gust, a ripple passing.
Wisps fly to the apex
The base is panting
My cage of ribs billows out again
I fill again.
In, out, in, out.
The cage is pliable though the limbs are stone.

How so?

I think I believe in an officer who exempts.
Sergeant right hemisphere has frozen your arm and your leg
But kept me warm and gusty.
A miracle of exemption

How selective illness is.

## Sensate

Funny thing, paralysis,
You feel nothing but you feel everything.
A symphony with the volume down

Heat comes at you, pain does, noise and light scream, in fact
Billions of nerves mumble, going nowhere, buzzing at you

You feel pleasure, though the world thinks you’re no longer really a woman
Or a man, with longings,
With a pulse in the groin
A concentric heat
A rippling depth
A moist suppleness

Meeting an other’s sensation
If they dare to meet yours
This half-body
This awkwardness,
This yearning for wholes

